# Interaction between the BAG1S isoform and HSP70 mediates the stability of anti-apoptotic proteins and the survival of osteosarcoma cells expressing oncogenic MYC

**DOI:** 10.1186/s12885-019-5454-2

**Published:** 2019-03-22

**Authors:** Victoria J. Gennaro, Helen Wedegaertner, Steven B. McMahon

**Affiliations:** Department of Biochemistry and Molecular Biology Sidney Kimmel Medical College, Thomas Jefferson University, Philadelphia, PA USA

**Keywords:** MYC, BAG1, HSP70, Apoptosis, Survival

## Abstract

**Background:**

The oncoprotein MYC has the dual capacity to drive cell cycle progression or induce apoptosis, depending on the cellular context. BAG1 was previously identified as a transcriptional target of MYC that functions as a critical determinant of this cell fate decision. The BAG1 protein is expressed as multiple isoforms, each having an array of distinct biochemical functions; however, the specific effector function of BAG1 that directs MYC-dependent cell survival has not been defined.

**Methods:**

In our studies the human osteosarcoma line U2OS expressing a conditional MYC-ER allele was used to induce oncogenic levels of MYC. We interrogated MYC-driven survival processes by modifying BAG1 protein expression. The function of the separate BAG1 isoforms was investigated by depleting cells of endogenous BAG1 and reintroducing the distinct isoforms. Flow cytometry and immunoblot assays were performed to analyze the effect of specific BAG1 isoforms on MYC-dependent apoptosis. These experiments were repeated to determine the role of the HSP70 chaperone complex in BAG1 survival processes. Finally, a proteomic approach was used to identify a set of specific pro-survival proteins controlled by the HSP70/BAG1 complex.

**Results:**

Loss of BAG1 resulted in robust MYC-induced apoptosis. Expression of the larger isoforms of BAG1, BAG1L and BAG1M, were insufficient to rescue survival in cells with oncogenic levels of MYC. Alternatively, reintroduction of BAG1S significantly reduced the level of apoptosis. Manipulation of the BAG1S interaction with HSP70 revealed that BAG1S provides its pro-survival function by serving as a cofactor for the HSP70 chaperone complex. Via a proteomic approach we identified and classified a set of pro-survival proteins controlled by this HSP70/BAG1 chaperone complex that contribute to the BAG1 anti-apoptotic phenotype.

**Conclusions:**

The small isoform of BAG1, BAG1S, in cooperation with the HSP70 chaperone complex, selectively mediates cell survival in MYC overexpressing tumor cells. We identified a set of specific pro-survival clients controlled by the HSP70/BAG1S chaperone complex. These clients define new nodes that could be therapeutically targeted to disrupt the survival of tumor cells driven by MYC activation. With MYC overexpression occurring in most human cancers, this introduces new strategies for cancer treatment.

**Electronic supplementary material:**

The online version of this article (10.1186/s12885-019-5454-2) contains supplementary material, which is available to authorized users.

## Background

The MYC oncogene encodes a transcription factor that is a central driver of cell-cycle progression in both normal settings and in cancer [1, 2]. An enduring paradox in the field is that MYC can also induce robust programmed cell death in some contexts [3]. As elevated MYC is a universal hallmark of human cancer, a great deal of effort has been expended in attempts to identify, and ultimately manipulate, the molecular events that convert the proliferative MYC program into an apoptotic program [4]. We previously identified a transcriptional target of MYC, termed BAG1, which is frequently overexpressed in human cancer and predictive of poor prognosis [5–8]. We formerly reported that blocking the induction of BAG1 by MYC converted the cellular response from proliferation to apoptosis [5].

The BAG1 gene encodes multiple protein isoforms that have a variety of highly diverse functions [9–12]. For example, BAG1 functions as a molecular chaperone responsible for proper protein folding and stability [13]. Additionally, BAG1 functions as a transcriptional cofactor for nuclear steroid receptors [14–18]. What remains unknown is which isoform-specific effector function of BAG1 is responsible for its potent pro-survival activity in the MYC pathway. The present study was undertaken to identify this effector function and subsequently assess the utility of targeting it to trigger the apoptosis of tumor cells expressing elevated levels of MYC.

We report here that a single isoform of BAG1, termed BAG1S, is responsible for the survival of tumor cells with elevated MYC. The BAG1S isoform has been linked to specific chaperone activities, in part through a physical association with the heat shock protein (HSP70). Point mutations in BAG1S that selectively disrupt its interaction with HSP70 eliminate the pro-survival function in MYC-expressing tumor cells. To gain an understanding of the specific biochemical events that mediated this pro-survival function, a proteome-wide screen was conducted that identified a unique set of pro-survival effector proteins [19]. Of clinical relevance, treatment of MYC-expressing tumor cells with a small molecule HSP70 inhibitor phenocopies the loss of BAG1S and selectively triggers the death of cells expressing oncogenic levels of MYC. Collectively, these findings broaden our understanding of the biochemical events that tumor cells trigger in order to survive in the context of otherwise lethal forms of stress.

## Methods

### Cell culture and plasmid expression

The human cell line U2OS MYC-ER was generously provided by Dr. Martin Eilers (University of Würzburg, Würzburg, Germany). Cells were cultured in Dulbecco’s modified Eagle’s medium (DMEM, Mediatech) supplemented with 10% fetal calf serum (FBS, Gemini Bio-Products) at 37 °C in 5% CO_2_.

BAG1L (50 kDa), BAG1M (46 kDa), and BAG1S (36 kDa) pcDNA3 expression plasmids were kindly provided by Dr. Graham Packham (University of Southampton, Southampton, UK). BAG1S C204A mutant was constructed by site-directed mutagenesis using QuikChange (Agilent Technologies). The ectopic BAG1 cell lines were generated by co-transfection of BAG1 plasmids and .25 fold of eGFP plasmid (Addgene). Transfection was performed using Continuum™ reagents according to the manufacturer’s protocol (Gemini Bio-Products). Cells were sorted by the Core Flow Cytometry Facility (Thomas Jefferson University, Philadelphia, PA) and cultured normally.

### Lentiviral infection and treatments

As indicated, cells were infected with lentiviral shRNA plasmids corresponding to BAG1 (NM_004323.4-1189s21c1) directed against the 3’UTR and control luciferase shRNA (SHC007) that were obtained from the TRC collection (Sigma-Aldrich). Lentiviral packaging plasmids (pCMV-R8.2 and pCMV-VSV-G) were cotransfected with shRNA vectors into 293 T cells (ATCC). Viral supernatants were collected, filtered, and added directly to target cells in the presence of 8 μg/ml polybrene (Sigma Aldrich). Cells were selected with with 8 μg/mL puromycin (Sigma-Aldrich) 24 h after infection.

The MYC-ER fusion protein was activated by adding 4-hydroxytamoxifen (4-OHT) (Sigma Aldrich) at a final concentration of 100 nM for indicated times. The HSP70 inhibitor MKT-077 (Sigma-Aldrich) was used at a final concentration of 10uM for 48 h.

### Apoptosis assays

To quantify apoptotic cell death, cells were collected by trypsinization and stained using the Annexin V PE-7AAD apoptosis detection kit (BD Pharmingen). Fluorescence was detected by flow cytometry CytoFlex LX Flow Cytometer (Beckman Coulter). 10,000 total events were collected and subsequently analyzed for the percentage of Annexin V-PE positive cells.

### Quantitative RT-PCR

Quantitative RT-PCR (RT-PCR) was performed by real-time analysis using the Step One Plus detection system (Applied Biosystems) and FAST SYBR GREEN PCR Master Mix kit (Applied Biosystems). RNA was isolated and purified using the Trizol extraction method (Invitrogen). cDNA was then generated using the High-Capacity cDNA Reverse Transcription Kit (Applied Biosystems).

### Immunoblotting and co-IP

Cells were harvested and lysed in E1A whole cell lysate buffer supplemented with protease inhibitor cocktail (Sigma-Aldrich). Immunoblotting concentration in lysates was determined using the bicinchoninic acid (BCA) assay and analyzed by SDS-PAGE using antibodies against BAG1 (generously provided by Dr. Graham Packham), HSP70 (Abcam, #ab2787), PARP (Cell Signaling, #9532), CASPASE-3 (Cell Signaling, #9662), RAF1 (R&D Systems, #MAB3585), XIAP (Cell Signaling, #2042), GCR (Cell Signaling, #3660), SLC7A6 (ThermoFisher, #PA5–30575), POLR1D (ThermoFisher, #PA5–30575), ACTIN (Santa Cruz, #sc-47,778), GAPDH (Abcam, #ab9485).

For protein-protein interaction studies, ∼750 μg of whole cell lysates (WCL) was used for immunoprecipitation (IP). BAG1 IPs were performed by incubating lysates with 10 μL Anti-BAG1 antibody (Abcam, #ab32109) or Rabbit IgG control (Abcam, #ab172730) for 16 h at 4 °C and precipitates captured using protein A/G beads (Santa Cruz).

### Proteomics LC-MS/MS

Experiment was performed in triplicate and all samples collectively subjected to proteomics analysis. Cells stably expressing vector control, BAG1S, or BAG1ΔS were infected with shRNA directed against BAG1 or luciferase (Vector-shLUC, Vector-shBAG1. BAG1S-shBAG1, BAG1ΔS-shBAG1). Five days post-infection, conditions were treated 100 nM 4-OHT for 12 h. Cellular extracts were standardized for protein quantity and run 0.5 cm into a 10% TRIS-Glycine gel (Invitrogen Novex). The sample lanes and a gel control were cut out and subsequently digested with trypsin. The digests were analyzed by LC-MS/MS on a Q Exactive HF mass spectrometer using an extended LC method. MS/MS spectra generated from the LC-MS/MS runs were searched using full tryptic specificity against the UniProt human database (www.uniprot.org; 10/01/2017) using the MaxQuant 1.6.1.0 program. “Match between runs” feature was used to help transfer identifications across experiments to minimize missing values. Protein quantification was performed using unique+razor peptides. Razor peptides are shared (non-unique) peptides assigned to the protein group with the most other peptides (Occam’s razor principle). False discovery rates for protein, and peptide identifications were set at 1%.

The output from MaxQuant identified 6225 total proteins. Common contaminants (e.g. keratins) and proteins identified by a single peptide (low-confidence identifications) were removed yielding 4915 remaining proteins. The abundance of a protein in a sample can be determined from the intensity i.e. the sum of the peptide MS intensities for the protein. To account for larger proteins generating more peptides, the intensity values were adjusted by dividing with the number of theoretical peptides for each protein (iBAQ intensity). In addition, the intensity values are also normalized (LFQ Intensity) to take into account the potential differences in sample loading. Normalization is done using the MaxLFQ algorithm [19]. The LFQ intensities were log2 transformed and then used for quantitative comparison. Student’s t-test (*p*-value) and fold change were calculated for binary comparison.

Conditions Vector-shBAG1, BAG1S-shBAG1, BAG1ΔS-shBAG1 were all divided by Vector-shLUC to normalize fold-change assessments to endogenous BAG1 levels. These standardized fold-change values were annotated knockdown (KD), BAG1S (S), and BAG1ΔS (ΔS) respectively. Proteins were sorted and hits with a *p*-value≤0.05 across all conditions were retained (309 proteins). To increase the stringency, proteins that did not have a ≥ |1.5| fold-change in the KD condition were discarded (153 proteins). Proteins were classified based on biological process characterized by UniProt. To visualize changes a heatmap was constructed using software described in *Nucleic Acids Research* (Babicki et al., Nucleic Acids Re, 2016).

The fold-changes of proteins in KD were compared with S and ΔS values to determine if reintroduction of either plasmid generated a partial rescue. Partial rescue was defined by an increase of ≥10% compared to KD.

### Statistical analysis

Data collected from at least three independent experiments are presented as mean ± standard deviation. Statistical testing was performed using SPSS with differences between two groups determined by a Student’s *t*-test. Significance is denoted in the figures as: ***P < 0.005; **P < 0.02; *P < 0.05.

## Results

### BAG1 protein required for blocking MYC-driven apoptosis

**Fig. 1:**
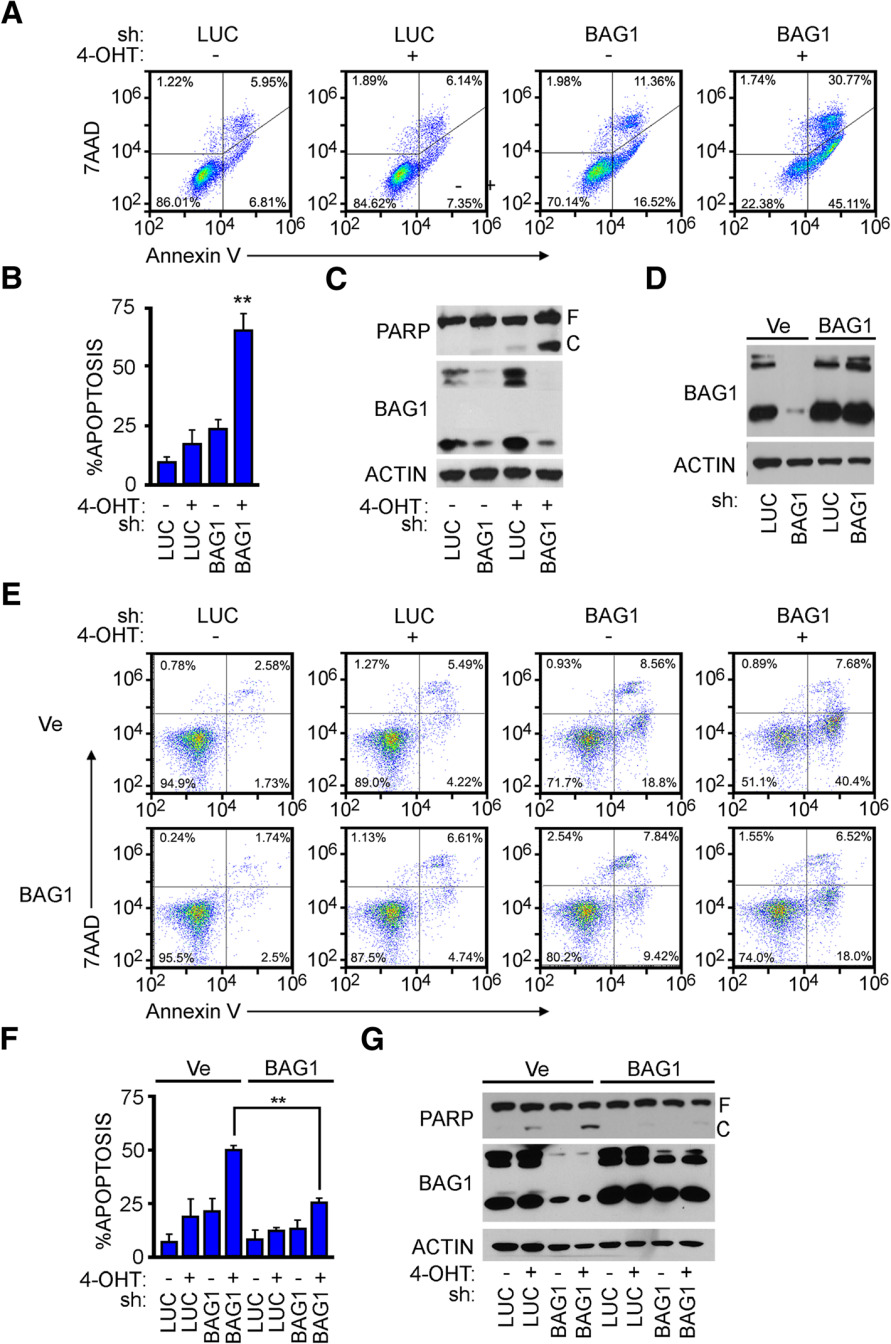
BAG1 protein sufficient to suppress MYC-driven apoptosis. **a** U2OS MYC-ER cells depleted of BAG1 via infection with an shRNA-encoding lentivirus directed against the 3’UTR or a luciferase shRNA control. Five days post-infection cells treated with ±100 nM 4-OHT for 48 h. Apoptosis measured by Annexin V-PE plus 7AAD DNA staining and quantified by FACS analysis. **b** Quantification of three experimental replicates representing average and standard deviation of cumulative early and late apoptosis based on population of Annexin V-PE positive cells. **c** PARP cleavage and BAG1 knockdown demonstrated by immunoblot (IB). F and C indicate full and cleaved species respectively. **d** U2OS MYC-ER cells transfected with vectors encoding BAG1 or empty vector, followed by endogenous BAG1 depletion. IB demonstrating efficient knockdown of endogenous BAG1 and retained ectopic BAG1 expression. **e** Cells from **d** cultured in ±100 nM 4-OHT for 48 h. Apoptosis measured by Annexin V-PE plus 7AAD DNA staining and quantified by FACS analysis. **f** Quantification of three experimental replicates representing average and standard deviation of apoptosis based on population of Annexin V-PE positive cells. **g** PARP cleavage and BAG1 knockdown demonstrated by IB. F and C indicate full and cleaved species respectively. **p < 0.02

To study MYC influence in malignant transformation, a system employing a conditional MYC-ER allele has been used extensively to mimic oncogenic levels of MYC function [20, 21]. Treatment with the estrogen analog 4-hydroxytamoxifen (4-OHT) activates this MYC-ER fusion protein and allows for induction of MYC activity (Additional file 1: Figure S1). Depending on the cellular context, MYC-ER cells display a robust MYC-dependent surge in either proliferation or apoptosis [22]. In human osteosarcoma U2OS MYC-ER cells where BAG1 induction was blocked, activation of MYC resulted in substantial apoptotic cell death [5]. As we reported previously, depletion of BAG1 followed by MYC activation via treatment with 4-OHT, resulted in a significant increase in apoptosis, as measured by either Annexin V staining or immunblotting for PARP cleavage (Fig. 1a, b and c). To verify that loss of BAG1 is the direct cause of the cell death observed with this shRNA construct, U2OS MYC-ER cells were generated to ectopically express the three major isoforms of the BAG1 protein. This exogenous BAG1 was resistant to knockdown by shRNA directed against the 3’UTR, yielding a system where all isoforms of endogenous BAG1 can be eliminated and the cells then rescued with individual isoforms (Fig. 1d). The combined ectopic expression of all three BAG1 isoforms significantly rescued the cell death marked by loss of endogenous BAG1 as observed by flow cytometry and apoptotic proteins to assess apoptosis (Fig. 1e, f and g). This established a platform for further dissecting the causal link between BAG1 and the suppression of MYC-driven apoptosis.

### The BAG1S isoform, but not BAG1L or BAG1M, is critical for survival of cells expressing oncogenic levels of MYC

**Fig. 2:**
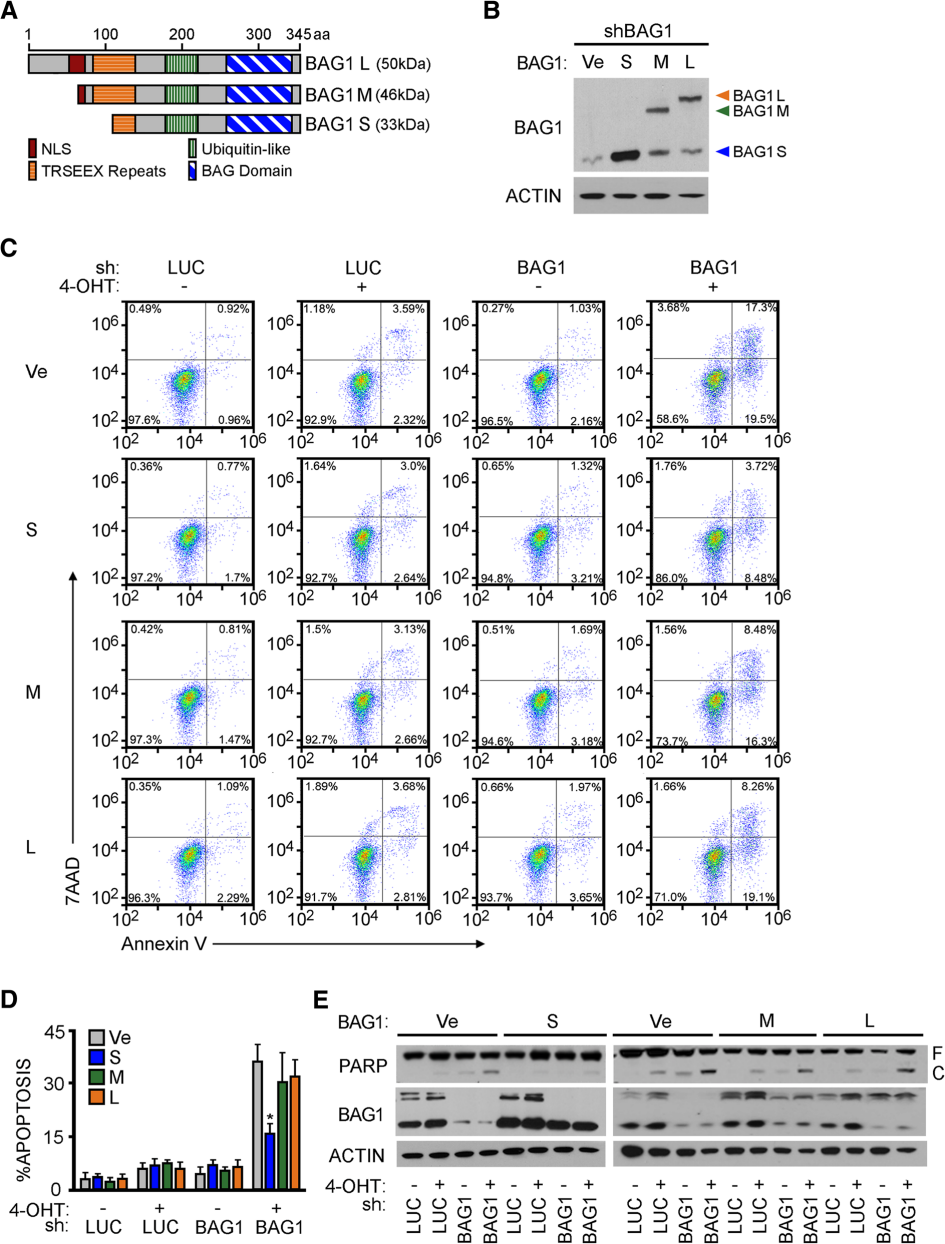
Individual expression of BAG1 isoforms revealed BAG1S critical to inhibit MYC-dependent cell death. **a** Schematic detailing the structure of human BAG1 isoforms L, M, and S. Amino acid positions shown on the top. Apparent molecular weights in kDa of protein products listed on the right. All isoforms share a ubiquitin-like domain, BAG domain, and complete (BAG1L and M) or partial (BAG1S) TESEEX hexapeptide repeats. Nuclear localization signal (NLS) domain present in BAG1L and partially in BAG1M. **b** U2OS MYC-ER cells transfected with vectors encoding distinct BAG1 isoforms L, M, S and an empty vector control. Cells depleted of endogenous BAG1 via infection with an shRNA-encoding lentivirus directed against the 3’UTR. IB demonstrating individual BAG1 isoform expression. **c** U2OS MYC-ER cells expressing distinct BAG1 isoforms depleted of endogenous BAG1 and treated with ±100 nM 4-OHT for 48 h. Apoptosis measured by Annexin V-PE plus 7AAD DNA staining and quantified by FACS analysis. **d** Quantification of three experimental replicates representing average and standard deviation of cumulative early and late apoptosis based on population of Annexin V-PE positive cells. **e** PARP cleavage and BAG1 expression demonstrated by IB from the same experiment but run on two separate gels. F and C indicate full and cleaved species respectively. *p < 0.05

BAG1 protein exists as multiple isoforms which originate from a single transcript of the human *BAG1* locus on chromosome 9 [9, 10]. A selection of different translation start sites generate the major BAG1 isoforms: BAG1L, BAG1M, and BAG1S [23]. The isoforms share a common carboxyl terminus, which includes ubiquitin-like and BAG domains [24]. However, the isoforms differ in the length of their amino termini. BAG1L and BAG1M contain 10 hexapeptide motif (TRSEEX) repeats, whereas BAG1S possess only four repeats. In addition, the extended amino terminus of BAG1L holds a nuclear localization signals (NLS) supporting its predominate localization to the nucleus [11]. Conversely, BAG1M and BAG1S are mainly detected in the cytosol [5, 9, 11, 23–25, 7–10] (Fig. 2a). Moreover, the different isoforms of BAG1 are linked to different effector functions. To assess the influence that different BAG1 isoforms have on MYC-dependent survival, U2OS MYC-ER cells were generated to exogenously express the individual isoforms. Depletion of endogenous BAG1 in each cell line demonstrated discrete rescue of either BAG1L, M, or S isoform expression when compared to cells transfected with vector control (Fig. 2b). As expected, apoptosis assays (both Annexin V staining and immunoblotting for and PARP cleavage) using the control cells demonstrated a substantial increase in cell death under conditions of MYC activation and endogenous BAG1 depletion. Neither of the larger isoforms, BAG1L or BAG1M, were sufficient to rescue survival in cells with oncogenic levels of MYC. However, under these conditions reintroduction of BAG1S significantly reduced the level of apoptosis (Fig. 2c, d and e). The selective ability of BAG1S in MYC-induced cells to repress death strongly suggested that the small isoform of BAG1 plays the key role in pro-survival function of BAG1 in this context.

### Interaction of BAG1S and HSP70 is essential for regulating the survival of cells with oncogenic MYC activity

Molecular chaperones of the 70kDA HSP70 family are involved in a multitude of cellular processes, including protein folding, protein translocation, and protein degradation [26–28]. To mediate these diverse functions, HSP70 cooperates with a variety of chaperone cofactors [29]. Specific cofactors either modulate the ATPase and peptide binding cycle of the chaperone or help direct HSP70 to specific proteins and subcellular compartments [30–32]. BAG1 is a component of the chaperone system that binds to the ATPase domain of HSP70 [33]. By making use of the ATP-binding domain rather than the substrate-binding domain, various other proteins can attach to the chaperone complex [15, 32, 34–36]. Previous studies indicated that HSP70 interacts with each of the BAG1 isoforms via the evolutionarily conserved BAG domain located in the carboxyl terminus and that these interactions enable different biochemical and biological activities of target proteins [37, 38].

**Fig. 3:**
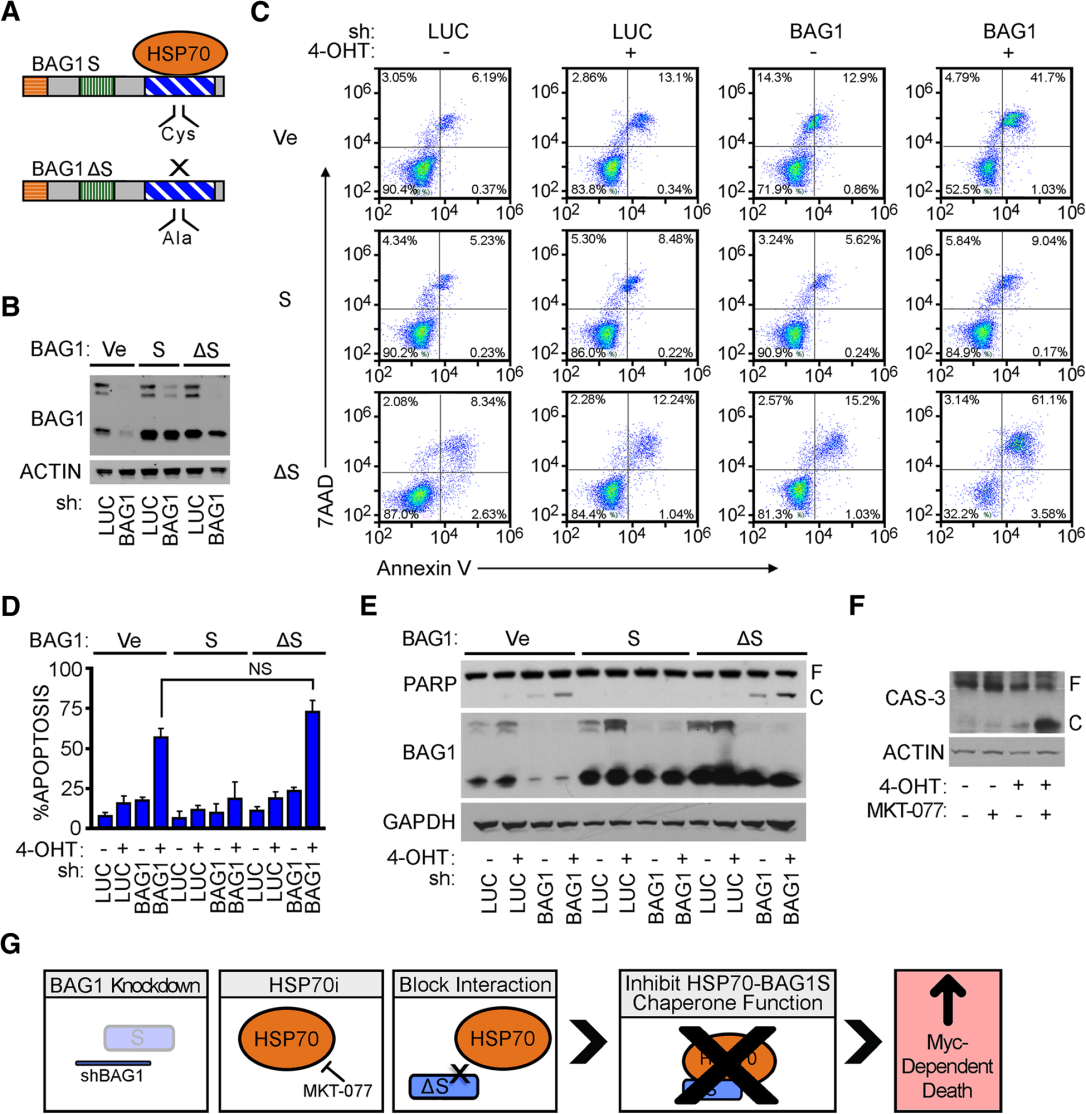
BAG1S mediated cell survival dependent on interaction with HSP70 chaperone. **a** Schematic demonstrating BAG1S interaction with HSP70 chaperone (top) and a mutant BAG1ΔS that retains BAG1S structure but prevents HSP70 binding via a single amino acid change in the BAG domain (bottom). **b** U2OS MYC-ER cells transfected with vectors encoding BAG1S, BAG1ΔS or empty vector, followed by endogenous BAG1 depletion or shLUC control. IB demonstrating efficient knockdown of endogenous BAG1 and retained ectopic BAG1S and BAG1ΔS expression. **c** U2OS MYC-ER cells expressing ectopic vector, BAG1S, or BAG1ΔS depleted of endogenous BAG1 via lentiviral infection. Five days post-infection cells treated with ±100 nM 4-OHT for 48 h. Apoptosis measured by Annexin V-PE plus 7AAD DNA staining and quantified by FACS analysis. **d** Quantification of three experimental replicates representing average and standard deviation of cumulative early and late apoptosis based on population of Annexin V-PE positive cells. **e** PARP cleavage and BAG1 knockdown demonstrated by IB. F and C indicate full and cleaved species respectively. **f** U2OS MYC-ER cells cultured with ±100 nM 4-OHT and 10uM MKT-077 HSP70 inhibitor for 48 h. CAS-3 demonstrated by IB. F and C indicate full and cleaved species respectively. **g** Model detailing three experimental methods used to interrupt HSP70/BAG1S chaperone complex activity. Limiting HSP70/BAG1S function via silencing BAG1, inhibiting HSP70, and preventing BAG1S binding HSP70 all resulted in increased MYC-dependent death

The HSP70 chaperone functions in part to eliminate mis-folded or mis-targeted proteins [39, 40]. Removal of corrupted proteins is crucial for cell viability because these proteins accumulate as non-specific aggregates which can become toxic to the cell [41, 42]. To determine if the anti-apoptotic function of BAG1S required the HSP70 chaperone complex, a mutant BAG1S was generated that prevented binding to HSP70 via a previously reported single amino acid change from a cysteine (Cys) to alanine (Ala) at residue 204 (C204A) (Fig. 3a) [43]. Stable U2OS MYC-ER cells expressing shRNA-resistant BAG1S mutant (BAG1ΔS), BAG1S, and vector control were developed and expression verified by depleting the cells of endogenous BAG1 (Fig. 3b). Immunoprecipitation (IP) of BAG1 confirmed that BAG1S, but not BAG1ΔS, was able to bind HSP70 (Additional file 2: Figure S2).

Apoptosis assays (both Annexin V staining and immunoblotting for PARP cleavage) demonstrated that the MYC-dependent cell death observed with loss of BAG1 is rescued by reintroduction of BAG1S but not BAG1ΔS (Fig. 3d, e and f). These findings suggest that BAG1S provides its pro-survival function by serving as a cofactor for the HSP70 chaperone complex.

Furthermore, obstructing chaperone function with the small molecule HSP70 inhibitor MKT-077 in combination with MYC activation resulted in robust cell death (Fig. 3g). Collectively, these data suggest that suppressing HSP70/BAG1 chaperone function by repressing expression/activity of either member separately or disrupting the interaction, rescues MYC-driven apoptosis. Stated more directly, MYC activation triggers the cell to become dependent on the cooperation and function of the HSP70/BAG1S chaperone complex for survival.

### Identification of a discreet set of pro-survival proteins regulated by the HSP70/BAG1 complex

**Fig. 4:**
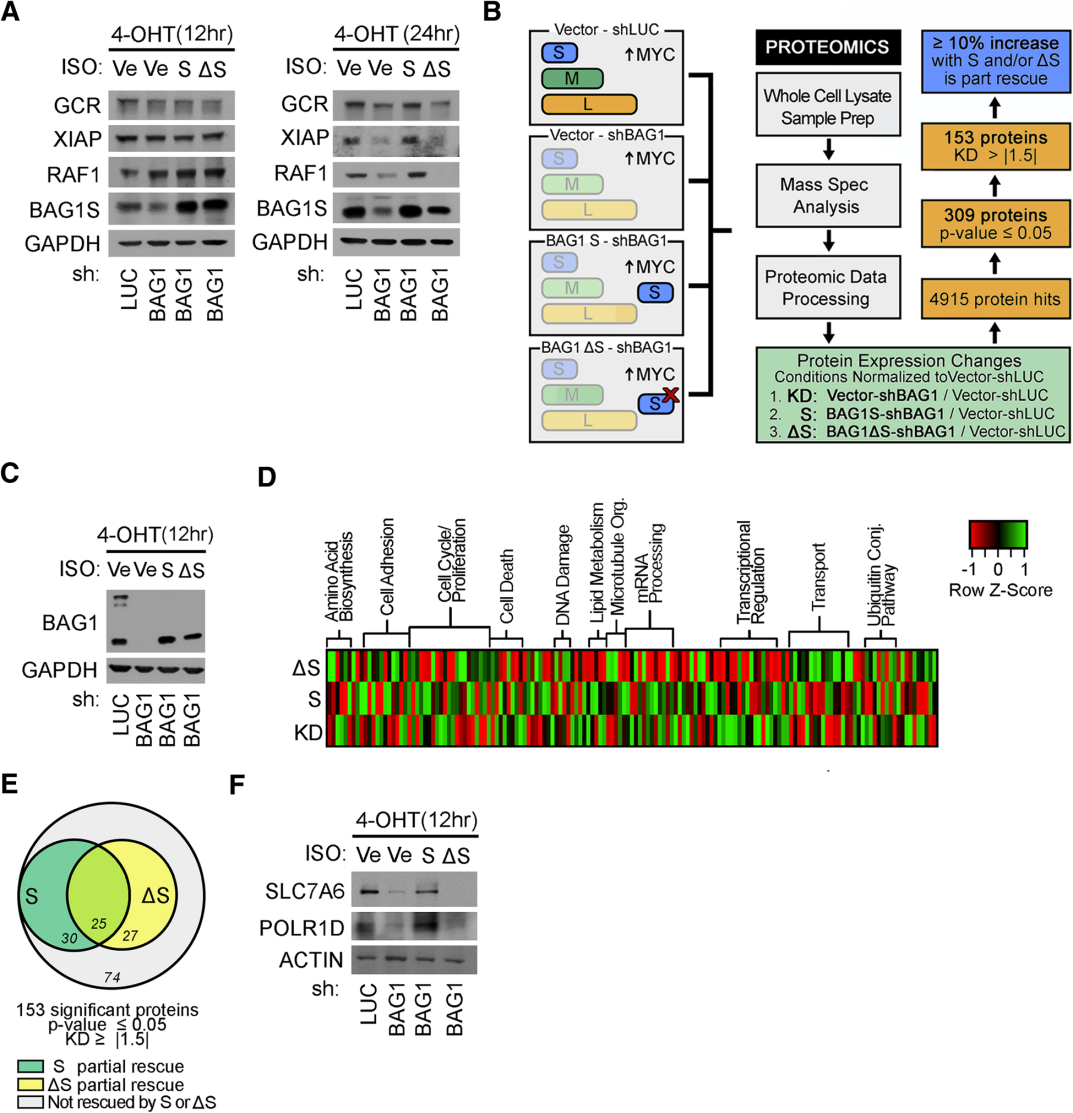
Proteomic analysis identified proteins upregulated in the presence of pro-survival HSP70/BAG1S complex. **a** U2OS MYC-ER cells expressing ectopic vector, BAG1S, or BAG1ΔS depleted of endogenous BAG1 protein. MYC activity induced for 12 or 24 h with ±100 nM 4-OHT treatment. Lysates analyzed via IB to detect changes in known HSP70 chaperone client proteins GCR, XIAP and RAF1. **b** Schematic of experimental conditions representing endogenous BAG1 (vector - shLUC), BAG1 knockdown (vector - shBAG1), BAG1S only (BAG1S - shBAG1), or BAG1ΔS only (BAG1ΔS - shBAG1) evaluated for differences in global protein levels. Proteomics analysis outlined with exclusion criteria for significant protein differences between samples. **c** Efficient knockdown of endogenous BAG1 and rescue of BAG1S and BAG1ΔS shown by IB for samples subjected to proteomics analysis. **d** Proteomic hits assessed based on schematic of compiled proteins with ≥|1.5| fold change in knockdown compared to control and *p* ≤ 0.05 across all conditions. Protein expression levels obtained for each sample indexed by specific protein and clustered by UniProt biological process classification. *P*-values representative of experimental triplicates submitted for proteomic assessment. **e** Venn diagram showing proteins partially rescued with reintroduction of BAG1S or BAG1ΔS. Increase of ≥10% constitutes a partial rescue. Overlapping proteins with BAG1S or BAG1ΔS indicative of proteins rescued by either ectopic protein. **f** Verification of proteomics via detection of BAG1S rescued targets SLC7A6 and POLR1D by IB.

BAG1 can modulate the ATP-driven activity of HSP70 and this regulation of chaperone function may provide a common mechanism to explain the diverse effects of BAG1 [44]. Although the protein refolding activity of HSP70 has received the greatest attention, chaperones can participate in a multitude of processes including protein translocation, stabilization, and degradation [26, 34]. BAG1 isoforms contain a ubiquitin-like domain (Fig. 2a) and can directly bind components of the ubiquitylation machinery and regulate client protein stability [12, 45, 46]. Glucocorticoid Receptor (GCR), X-linked Inhibitor of Apoptosis Protein (XIAP), and Rapidly Accelerating Fibrosarcoma 1 (RAF1) have been previously described as clients of HSP70 chaperone activity that function in part to activate pro-survival pathways [13, 43, 47]. To assess the dependence of MYC and BAG1S on these established HSP70 client proteins, we activated MYC for 12 or 24 h (as indicated) in context of endogenous BAG1, loss of BAG1 or singularly expressed BAG1S, both unmodified and mutated to prevent interaction with HSP70 (BAG1ΔS) (Fig. 4a). With 12 h of oncogenic MYC activity, chaperone targets were not substantially affected. However, after 24 h of MYC induction, client proteins were stabilized in conditions where the HSP70/BAG1S chaperone complex remained intact (Fig. 4a). Importantly, the stabilization of pro-survival proteins GCR, XIAP, and RAF1 by sustained HSP70/BAG1S expression provides a potential mechanisms by which BAG1S-dependent survival can be controlled.

**Fig. 5:**
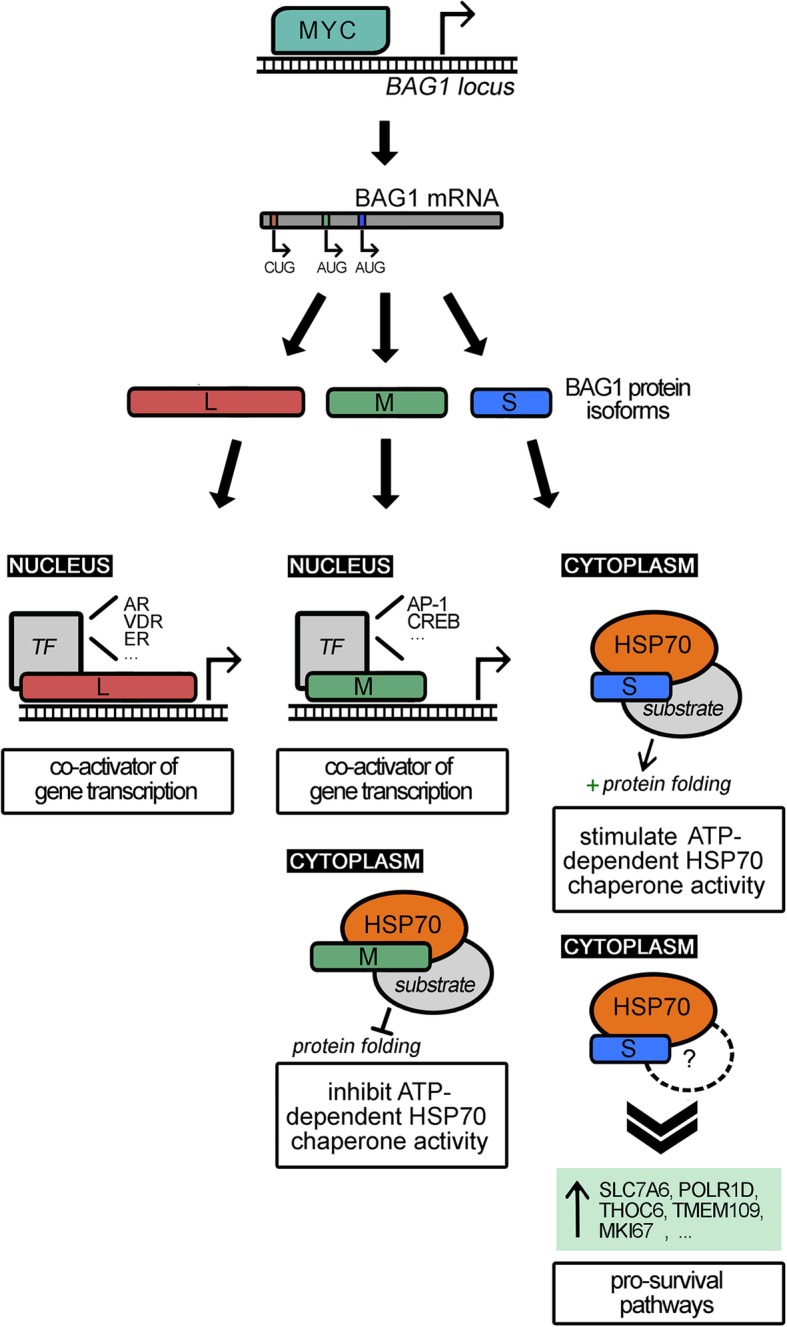
Proposed model of MYC-mediated BAG1 regulation and distinct functions of BAG1 isoforms. MYC activates gene transcription of *BAG1* which produces BAG1 mRNA with three translation start sites that encode distinct protein isoforms BAG1L, M, and S. Nuclear BAG1L and BAG1M cooperate with transcription factors to regulate gene expression. Cytoplasmic BAG1M and BAG1S interact with HSP70 to modulate chaperone activity. As reported here, HSP70/BAG1S complex upregulates an array of proteins and inhibits MYC-driven apoptosis

In an effort to evaluate proteins that could contribute to BAG1S-driven pro-survival with early oncogenic MYC activation, an unbiased, comprehensive proteomics screen was performed (Fig. 4c). Three experimental replicates were subject to LC-MS/MS analysis to identify consistent and significant protein alterations. A visual representation of the proteomic analysis scheme is provided in Fig. 4b, and the procedure is further described in the Methods. In brief, levels of individual proteins from conditions with loss of BAG1 (KD), ectopic BAG1S (S), and ectopic BAG1ΔS (ΔS) were compared to levels in cells expressing endogenous BAG1 (shLUC control). This allowed normalization of protein fold-changes. Proteins that had a significant *p*-value (≤0.05) across all conditions and a change in expression of ≥|1.5| in knockdown (KD) were further assessed. The output proteins were classified based on biological process characterized by UniProt and differential expression of proteins in each condition was visualized via heatmap (Fig. 4d). Of the 153 proteins that whose levels were controlled by BAG1, 56 were at least partially rescued by reintroduction of BAG1S, and 30 of those were specific to BAG1S and not HSP70-interaction deficient mutant BAG1ΔS (Fig. 4e). Several of the proteins rescued by BAG1S and not BAG1ΔS are presented in Table 1. Empirically assessing protein expression for two of the BAG1S-specific rescued proteins, Solute Carrier Family 7 Member 6 (SLC7A6) and RNA Polymerases I Subunit D (POLR1D), verified the results of the screen (Fig. 4g). Of the 30 proteins whose regulation by BAG1S correlates with protection from MYC-induced apoptosis, nearly half have been previously associated with survival [48–62]. Furthermore, the majority of identified proteins were formerly linked with MYC (Table 1) [52, 58, 63–70]. BAG1 expression is a biomarker of poor prognosis, presumably because it activates a pro-survival pathway that tumor cells need in order to proliferate. By characterizing additional members of this pro-survival process we are closer to identifying methods to restore MYC-driven apoptosis. Importantly, several of the proteins identified are candidates for therapeutic targeting based on existing drugs [71–74]. Collectively, this adds to the existing knowledge of BAG1-mediated activity, which is summed and graphically represented in Fig. 5.

**Table 1:**
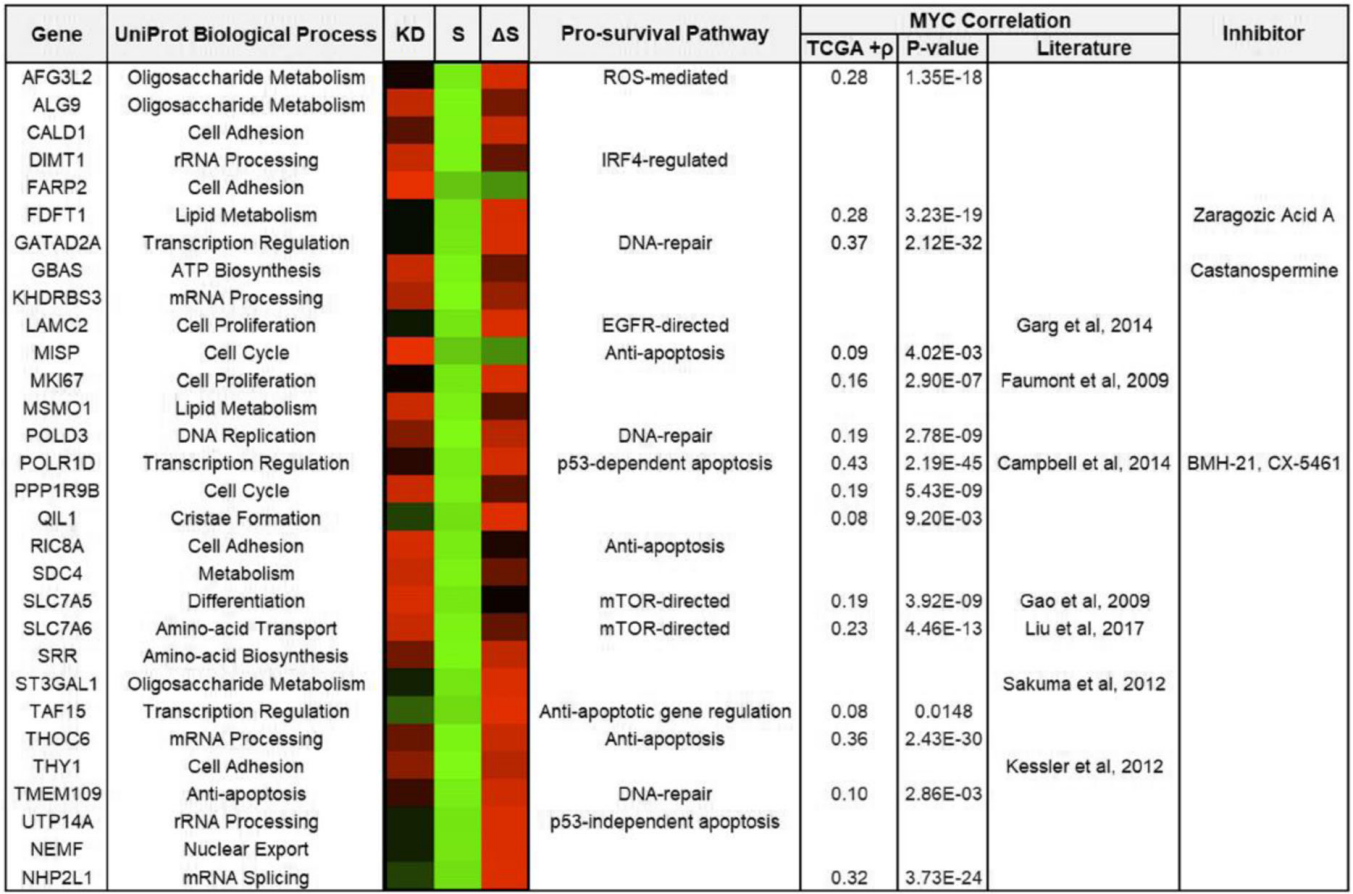
Classification of HSP70/BAG1S Clients

## Discussion

MYC is overexpressed in human cancer at an unparalleled frequency [4]. This is consistent with the ability of MYC to drive cell cycle progression, a phenotypic hallmark of cancer [1, 2]. Ironically, MYC can also drive apoptotic death, in some cellular context [75]. The ability to rationally reprogram MYC function in tumors, from cell cycle progression to apoptosis, could provide an attractive therapeutic strategy. Among the biochemical mechanisms that control the switch between cell cycle and apoptosis is MYC’s ability to directly activate transcription of the pro-survival gene BAG1 [5]. BAG1 is often overexpressed in human cancer and elevated BAG1 levels is associated with poor prognosis [6–8].

While identification of BAG1’s role in this process was a conceptual advance, BAG1’s functions are exceedingly broad, thus presenting a barrier to identification of specific nodes for targeting. Adding to the complexity, the BAG1 protein is expressed as several distinct isoforms that have disparate activities and subcellular localizations [9, 16, 23–25, 76]. As examples, BAG1L impacts gene regulation by altering the activity of key nuclear hormone receptor (NHR) transcription factors, including the androgen receptor (AR), vitamin D receptor (VDR), Activator Protein 1 (AP-1), and Estrogen Receptor (ER) [18, 77–82]. Similarly, BAG1M cooperates with transcription factors like AP-1 and cAMP Response Element-Binding Protein (CREB) to mediate target gene regulation [17, 18, 36].

Alternatively, BAG1M and BAG1S interact with HSP70 and regulate an array of molecular targets within the cytoplasm [45]. The different amino-termini of the distinct cytoplasmic BAG1 isoforms dictate the consequences of the HSP70-mediated folding events of accessory proteins [9]. BAG1M inhibits the HSP70-mediated refolding of the non-native polypeptide substrates, while BAG1S stimulates HSP70 chaperone activity [45]. The antagonistic influence of BAG1M and BAG1S on HSP70 regulation of client proteins impacts different pathways involved in cell proliferation, apoptosis, and stress response.

We demonstrate here that it is specifically the S isoform of BAG1 that provides the survival function in MYC overexpressing tumor cells. Using both genetic and pharmacological approaches, we further demonstrate that it is the HSP70/BAGS1 chaperone complex that provides the survival function. Via a proteomic approach we identify and then validate a set of specific proteins and pathways controlled by this HSP70/BAG1S chaperone complex. Many of the proteins identified are positively correlated with MYC and have reported roles in survival pathways. The identification and further characterization of these clients of the HSP70/BAG1 complex provides the initial step towards targeted therapies that can convert MYC from a pro-tumorigenic oncogene to a pro-apoptotic tumor suppressor.

## Conclusion

The small isoform of BAG1, BAG1S, in cooperation with the HSP70 chaperone complex, promotes cell survival in MYC overexpressing tumor cells. We identify specific proteins controlled by the HSP70/BAG1S chaperone complex that contribute to MYC-driven pro-survival in cancer cells. The identification of these HSP70/BAG1 chaperone clients introduces new targets that could be therapeutically exploited to disrupt the survival of tumor cells driven by MYC activation.

## Additional files

Additional file 1: **Figure S1.** MYC-ER simulates oncogenic MYC activity in U2OS cancer cells. Activation of MYC-ER in U2OS cells with 4-OHT treatment demonstrated characteristic loss of endogenous MYC expression and simultaneous mRNA induction of known MYC targets BAG1, CAD and CCND2. (PDF 75 kb)

Additional file 2: **Figure S2.** Verification of HSP70 interaction with BAG1S and not BAG1ΔS. Immunoprecipitation of BAG1 in U2OS MYC-ER cells with depleted endogenous BAG1 and sustained ectopic expression of either BAG1S or BAG1ΔS demonstrated HSP70 interaction with BAG1S, but not BAG1ΔS. (PDF 375 kb)

## Abbreviations

4-OHT: 4-hydroxytamoxifen
Ala: Alanine
AP-1: Activator protein 1
AR: Androgen receptor
CREB: cAMP Response Element-Binding Protein
Cys: Cysteine
DMEM: Dulbecco’s Modified Eagle’s Medium
ER: Estrogen receptor
GCR: Glucocorticoid Receptor
HSP70: Heat Shock Protein
IP: Immunoprecipitation
KD: Knockdown
MYC-ER: MYC-estrogen receptor fusion protein
NHR: Nuclear hormone recptor
NLS: Nuclear localization signals
POLR1D: RNA Polymerases I Subunit D
RAF1: Rapidly Accelerating Fibrosarcoma 1
SLC7A6: Solute Carrier Family 7 Member 6
TRSEEX: Hexapeptide motif
VDR: Vitamin D receptor
XIAP: X-linked Inhibitor of Apoptosis Protein
