# Supplementary material for: Interaction between the BAG1S isoform and HSP70 mediates the stability of anti-apoptotic proteins and the survival of osteosarcoma cells expressing oncogenic MYC
Source: BMC Cancer. 2019 Mar 22;19:258. doi: 10.1186/s12885-019-5454-2 (PMC6429775; doi:10.1186/s12885-019-5454-2)
Supplement: Supplementary file 1 — Figure S1. MYC-ER simulates oncogenic MYC activity in U2OS cancer cells. Activation of MYC-ER in U2OS cells with 4-OHT treatment demonstrated characteristic loss of endogenous MYC expression and simultaneous mRNA induction of known MYC targets BAG1, CAD and CCND2. (PDF 75 kb) [file 12885_2019_5454_MOESM1_ESM.pdf]

Figure S1. MYC-ER simulates oncogenic MYC activity in U2OS cancer cells

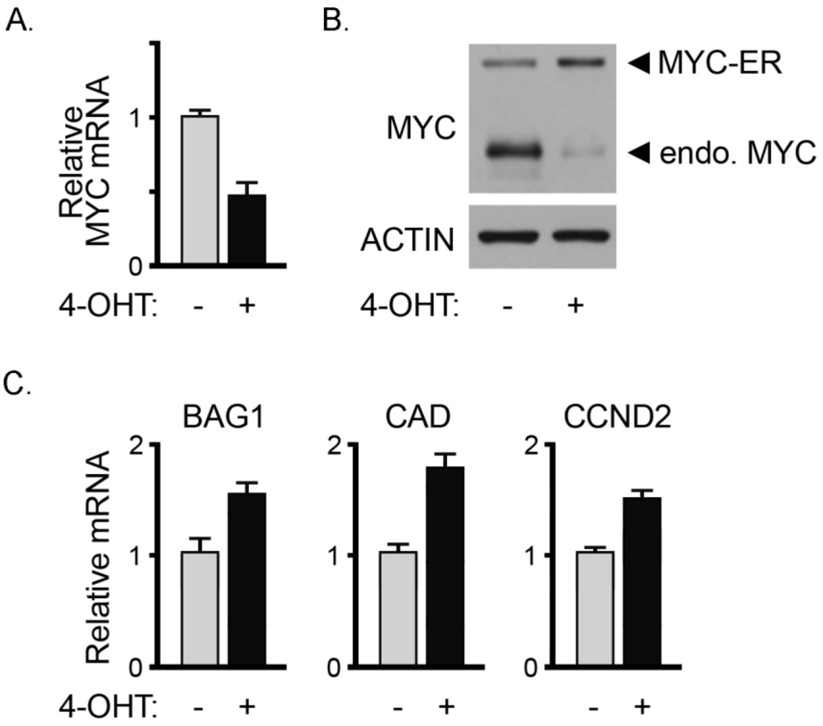

Figure S1. MYC-ER simulates oncogenic MYC activity in U2OS cancer cells

U2OS cells engineered to express MYC-ER were treated with 100nM 4-OHT for 48h to induce MYC activity. (A) MYC mRNA was quantified using RT-PCR and demonstrated a reduction in endogenous MYC transcription upon MYC-ER activation. (B) Western blot depicted loss of endogenous MYC protein and increase in MYC-ER fusion protein after 4-OHT treatment. (C) RT-PCR assessment of known MYC target genes BAG1, CAD, and CCND2 revealed induction of transcription after MYC-ER activation.
