# Supplementary material for: Interaction between the BAG1S isoform and HSP70 mediates the stability of anti-apoptotic proteins and the survival of osteosarcoma cells expressing oncogenic MYC
Source: BMC Cancer. 2019 Mar 22;19:258. doi: 10.1186/s12885-019-5454-2 (PMC6429775; doi:10.1186/s12885-019-5454-2)
Supplement: Supplementary file 2 — Figure S2. Verification of HSP70 interaction with BAG1S and not BAG1ΔS. Immunoprecipitation of BAG1 in U2OS MYC-ER cells with depleted endogenous BAG1 and sustained ectopic expression of either BAG1S or BAG1ΔS demonstrated HSP70 interaction with BAG1S, but not BAG1ΔS. (PDF 375 kb) [file 12885_2019_5454_MOESM2_ESM.pdf]

Figure S2. Verification of HSP70 interaction with BAG1S and not BAG1ΔS

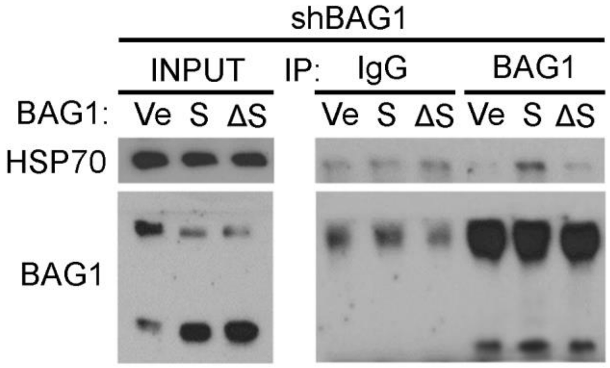

Figure S2. Verification of HSP70 interaction with BAG1S and not BAG1ΔS

U2OS MYC-ER cell lysates subjected to a BAG1 IP under non-denatured conditions using A/G beads. Precipitates probed for HSP70 to detect interaction with BAG1S and lapsed interaction with BAG1ΔS. Confirmation of pull-down and sufficient knockdown of endogenous BAG1 was observed by probing for BAG1.
